# Digging deeper in Shanghai: towards a ‘mechanism-rich’ epidemiology

**DOI:** 10.1093/inthealth/ihz057

**Published:** 2019-10-30

**Authors:** Jie Li, Nick Manning, Andrea Mechelli

**Affiliations:** 1 School of Tourism Management, Sun Yat-Sen University, Tangjiawan Town, Zhuhai, Guangdong, China; 2 Department of Global Health & Social Medicine, School of Global Affairs, King’s College London, 30 Aldwych, London WC2B 4BG, UK; 3 Department of Psychosis Studies, Institute of Psychiatry, Psychology & Neuroscience, King’s College London, De Crespigny Park, London SE5 8AF, UK

**Keywords:** ecological assessment, ethnography-informed survey, mental health, migrant, mobility, smartphone app

## Abstract

**Background:**

There are very few close-up sociological or anthropological data informing epidemiological and psychiatric research design and/or contributing to our understanding of the relationship between mental health and specific forms of urban life. Furthermore, research on the relationships between urbanicity and mental disorder has paid little attention to the global diversity of urban experience, such as in cities in China, India and Brazil.

**Methods:**

Two innovative methods can be employed to unveil the diversified urban experience of migrants in China, i.e. an ethnography-informed sociological deep surveying instrument and an ecological momentary assessment with a smartphone app. This article introduces the design and pilot survey of these new instruments towards a ‘mechanism-rich’ epidemiology.

**Results:**

The ethnography-informed survey instrument enabled us to include some of the issues from the ethnography and successfully ‘dig deeper’ into respondents’ social experience. The pilot of the smartphone app serves as ‘proof of principle’ that we can recruit respondents in Shanghai, and that we can receive and use the data.

**Conclusions:**

Both of these pilots have demonstrated good feasibility for studying mobility, urban life and mental health. Our next steps will be to extend the Shanghai sample, to use the app in Sao Paulo and Toronto and then hopefully in India and Africa.

## Introduction

Since the late nineteenth century, urban transformation has been associated with mental illness.^1^ There have been longstanding and consistent findings on the association between city living and poor mental health in psychiatric epidemiology.^2–5^ But there are questions around the specificity of the relationship. Why does mental illness cluster in cities? What are the relationships of urban poverty, deprivation, overcrowding, social exclusion, racism and violence to mental distress? What biological and sociological mechanisms might be at stake, and which have continued well into the twenty-first century?^6^ Research has identified key phenomena that tie mental illness and the social lives of cities together: among the factors most consistently identified are migration, density and stress: migrants into cities bear a disproportionately large share of the burden of urban mental illness^7^; dense living conditions exacerbate the problem^8^; and the general stress and precarity of urban living create the psychosocial basis for the development of clinical problems.^9^

In the early years of China’s market reform, mental healthcare was only provided in psychiatric hospitals. Since the 2000s, there has been a gradual national mental health service reform that aimed to include mental health policy in the national public health programme. However, the focus of mental health policy was primarily on disorders associated with violent or socially disruptive behaviours, as social harmony and stability was a priority concern for the Chinese government.^10^ The Chinese government incorporated major mental disorders into the public healthcare scheme in 2009. The National Mental Health Law of 2013 provides the legal basis to protect the legitimate rights and interests of people with mental disorders. In the meantime, there has been a growing popular interest in psychological counselling, but it was essentially a ‘middle class thing’, centred around urban professionals. Migrants are largely left out in China’s ‘psycho-boom’, compounded by a general official negligence of migrant mental health matters (L. Richaud and A. Amin, manuscript submitted).^11^ The situation for those suffering mental distress is made worse by stigma toward people with mental disorders embedded in Chinese traditional culture.^12^ In contemporary China, where development is intrinsically related to migration from the countryside into the cities, effective development of policies and governance requires greater understanding of the links between urban stress, precarity and mental disorder. Unrecognized and untreated mental disorder is a key factor in casting individuals and families further into poverty and social exclusion.

Much research is now underway to refine our understanding of the biological mechanisms through which urban life gets translated into clinical symptoms.^2^ But there is a series of interdisciplinary and comparative questions which need to be addressed if mental health scholars and policymakers are to intervene effectively: first, we know that the relationship between urbanicity and mental illness results from a complex intertwining of biological, psychological and sociological factors. But we have only a very limited understanding of how, precisely, they are related. Partly because of the estrangement of the qualitative social sciences from the psychiatric and epidemiological sciences,^13^ and despite the conceptualization of the biosocial body in social science as varied in terms of ‘local biologies’^14^ or ‘multiple bodies’,^15^ there are very few close-up sociological or anthropological data informing epidemiological and psychiatric research design and/or contributing to our understanding of the relationship between mental health and specific forms of urban life as such. Second, since it emerged in the nineteenth century, research on the relationships between urbanicity and mental disorder has focused on the industrial cities of North America and Europe^16^ and has paid strikingly little attention to the global diversity of urban experience. In particular, despite the wide recognition that we are in the midst of another period of significant urbanization whose prime exemplars are in Asia and in Latin America, remarkably little is known about the relationship between mental health and the rapidly expanding mega-cities of our century in countries such as China, Brazil and India (Andrade et al.^17^; Andrade et al., this special issue^18^). Therefore, we aim to unveil the diversified urban experience of migrants in Shanghai, China, with innovative methods, including ethnography, an ethnography-informed sociological deep surveying instrument and an ecological momentary assessment with a smartphone app. These methods are introduced below.

The ethnographic study has focused on the lived experiences of migrants, migrant households and migrant neighbourhoods in Shanghai (Lisa and Ash, 2019).^19^ This is a focus not only on people, but also on the situations through which people move and create their urban lives: our sampling frame therefore includes both people and their situations, such as their domestic places, work settings, service consumption (healthcare, education and housing) and locations for friendship and conviviality. This method draws out smaller scale, less visible aspects that comprise the physical and technical elements of city life, as well as the culture, economy and family/personal circumstances which create or mitigate. While epidemiological research acts according to predefined variables of urban life on large populations, the ethnography method here is interested in what urbanicity and its qualities actually are. It consists of a ‘deep sampling’ strategy, in which a large amount of thick, qualitative data is collected from a relatively small number of participants at a number of ethnographic sites. The ethnographic work has been concentrated on both the suburban ‘edges’ and the centre of Shanghai (L. Richaud and A. Amin, manuscript submitted).^11^ One key element that the ethnography has been focused on is ‘urban stress’. The concept of urban stress remains elusive; however, it often refers to daily hassles or stresses that are typical of dense urban environments.^20,21^ In our study, we conceptualized urban stress in migrants in terms of difficulties that disrupt their life in the city, such as accommodation being demolished for urban renewal or getting their children admitted to local schools. These are captured through observation and communication with participants. The key aspect we found were the ways in which migrants successfully manage this stress through a number of social actions, rather than the ways in which they react through mental distress/disorder, including slowing down the impact of change by maintaining routines, shared public discussion about problems to generate a kind of local solidarity, making small efforts to retain aspects of their lives in the hope that things will improve, filling ‘empty’ time through card games and by the use of public libraries, mobile phone ‘escapism’ to avoid current pressures, mutual mental support and ideas of happiness, re-stating the opportunities for a better life in Shanghai and continuing to hope for it.

Findings from the ethnographic work are in part designed to inform the compilation and trial of a sociological deep surveying instrument for mapping migrant mental health in Shanghai. The purpose of the pilot survey is to figure out whether we can survey the situations revealed by ethnography directly in any way. A traditional form of collaboration between epidemiology and anthropology involves using ethnographic insight to better develop questionnaires for quantitative surveys, primarily by improving the wording and social suitability of questions.^22^ Following Béhague, Gonçalves and Victora,^22^ we took this form of collaboration further by using ethnographic insight to introduce unexpected questions into our survey that would help us refine our theoretical and interpretive understanding of rural–urban migration and mental health. The survey not only measured the experience of urban stressors and the concomitant expression of psychiatric symptoms, but also measured and reported on forms of social experience (including access to resources, forms of family life and highly localized forms of conviviality and experience) emergent from the ethnographic account of the ‘migrant street’ in Shanghai. It included conventional epidemiological categories used in national and international comparisons, but it also covered specific migrant experience, particularly why they migrate to the city, how they perceive the city and their lives, and how they perceive and cope with the specific stress related to migration; we draw upon the close-up ethnographic work, which could have implications on their mental well-being.

The survey included the following items: general demographics; aspects of daily life in Shanghai; aspects of work; commuting; aspects of social life; aspects of housing; neighbourhood; attitudes to Shanghai; aspirations; SCL-10 (symptom checklist); K6 (Kessler psychological distress); PSS-10 (perceived stress scale); adverse childhood experience scale; workplace social capital scale; neighbourhood social capital scale; and social support scale. It is hoped that the integration of an ethnographic approach within the thematic content of the survey will help us advance our theoretical and explanatory understanding of migrants’ mental health in mega-cities.

The smartphone app, ‘Urban Mind’, examines the relationship between the built urban environment and mental well-being, using ecological momentary assessment (EMA). EMA is a technique that involves multiple sampling of participants’ current experiences and behaviours in real-world environments. It allows the assessment of particular events in participants’ lives at periodic intervals via random time sampling; this minimizes recall bias, maximizes ecological validity and provides insight into dynamic changes within real-world contexts. A more detailed description of the app will be provided in the New survey method through smartphone app. Following this Introduction, next we present data and findings from the pilot survey, then introduce how the app works in detail and present some preliminary data. Detailed findings from the ethnography are presented in another paper in this special issue.

## Ethnographic-informed pilot survey

The pilot survey was conducted in January and July 2018, both in suburban areas and in the central city of Shanghai. A convenience sampling strategy was adopted. The survey sites included one residential community for a migrant population in Songjiang and other public places such as streets, parks or small shops, restaurants and bubble tea stores where migrants from the rural area are most likely to work. A total of 135 respondents were surveyed and, after removing those with missing data, 102 respondents were included in this study. The sociodemographic structure of the sample is summarized in Table 1; 70% of respondents had rural hukou, meaning that they belong to the category of rural-to-urban migrants, and the other 30% were mostly from small towns or counties. The term hukou (household registration) specifically refers to two aspects of population registration. Under the hukou system, each individual was registered in one place of residence and was categorized as a rural or urban resident. In the socialist era, people with an urban hukou were entitled to social resources (e.g. housing, education and healthcare) that were not available to those with a rural hukou, and this constrained rural to urban migration. While a series of reforms of the hukou system have been conducted since China’s market reform, those without a local urban hukou are still deprived of full citizenship rights, particularly in first-tier cities such as Beijing and Shanghai.^23^

**Table 1.** Sociodemographic structure and family arrangement of the sample

Variable	Percentage	Variable		Number of cases
Gender				
Male	52%	Number of children	1	34
Female	48%		2	14
Age, y				
19–29	55%		3 or more	3
30–39	27%	Location of children	With me	21
40–49	13%		With my spouse	16
50–59	5%		With my parents/in-laws	12
Education				
Middle school or less	36%		Other	2
High school or more	64%	Location of spouse	Also in Shanghai	35
Marital status				
Not married	47%		In another city	7
Married	53%		In hometown	14
Income (yuan)				
<5000	40%	Housing	Dormitories	37
5000–10 000	40%		Informal rental housing	46
>10 000	20%		Formal rental housing	7
Hukou				
Rural	70%		Self-owned	10
Urban	30%		Other	2

### Perception of the city of Shanghai

In the survey, we asked respondents why they came to Shanghai. About 40% stated that they came to Shanghai because of more job opportunities, 26% chose Shanghai as their migration destination because they already had friends or relatives in Shanghai and 18% came to Shanghai to earn a higher income. Other answers included ‘to change an environment’, ‘have business connections in Shanghai’ and ‘just follow my heart’. It is interesting to note that none of the respondents chose the option ‘for better public service such as healthcare or transport’, despite the common perception of Shanghai as a city that provides the best public service in China. The reason for this could be that without a Shanghai hukou, rural migrants are excluded from many public services in the city. Migrants took a very pragmatic view about what they could get from the city, which is work experience and money. In other words, they accumulate their human and economic capital in Shanghai so that they can start a better life in a smaller city or county later on. This is also reflected in the answers to the question ‘Do you plan to leave Shanghai?’, to which only 18% indicated that they had no intention to leave Shanghai in the future. Some migrants had a clear plan, such as to return to their hometowns in 1 or 2 y, get married, open their own shop, or to take care of their wives and children; others did not have a specific leaving plan at the moment but were sure that they would leave eventually when they or their children reached a certain age.

**Figure 1.**
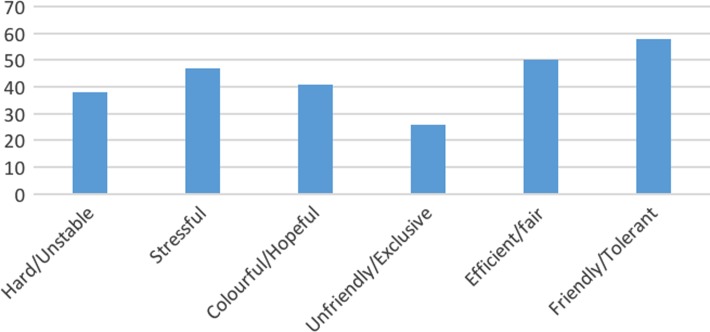
Respondents’ perceptions of their lives in Shanghai and the city of Shanghai. Participants were asked to choose three items at most to describe their life in Shanghai and their perception of the city of Shanghai.

Regarding the question of whether they liked Shanghai or not, about three-quarters of participants gave an affirmative answer that they did like Shanghai, despite the stressful urban life. When asked the reason why they liked Shanghai, the answers given can be roughly summarized into two categories. The first were personal development-oriented: ‘It offers more opportunities for personal development’; [I can] ‘broaden my view, I can learn more new things’; and [there are] ‘more job opportunities and resources’. The second were lifestyle-oriented, for example, some respondents indicated that they liked the variety of night life and different kinds of delicious food available in Shanghai. A few respondents also mentioned that they like the cosmopolitan and international feel of the city, and that the city is safer and more civilized than their hometowns. In particular, at least five respondents’ first reaction was that ‘I like the fast-paced rhythm in Shanghai’ or [I] ‘got used to the fast-paced life’. It is worth noting that a rapid pace of life in a modern cosmopolitan city is often linked with heightened stress and health problems,^20,24^ but some rural migrants actually enjoy the busy lifestyle in a mega-city. Perhaps to them, a fast-paced rhythm means hope of a better future and a more fulfilling life. Nonetheless, some respondents indicated they did not like Shanghai as life in Shanghai was ‘too stressful’, ‘very tiring’ and they had a sense of ‘being excluded’ (Figure 1). About one-third of participants indicated that their lives in Shanghai can be characterized as ‘hard’ and/or ‘precarious’, and almost half of respondents admitted that their lives were ‘stressful’. Among those who perceived their lives in Shanghai as stressful, 40% also stated that their lives were ‘colourful’ and/or ‘hopeful’. More respondents held a positive rather than a negative view towards the city. A quarter of respondents perceived Shanghai as unfriendly or exclusive, and one-fifth perceived the city as depressing. By contrast, 57% respondents perceived Shanghai as friendly and/or tolerant and 50% of respondents perceived Shanghai as efficient and/or fair.

### Stress in the mega-city of Shanghai

We also asked respondents what they perceived as the most stressful aspect of living in Shanghai. The Chinese term for stress is ‘yali’. Similar to stress, the word also means pressure and emotional strain or tension, but it does not have the same meaning of physiological reaction or disturbance that stress does. In this article, stress refers to emotional tension or psychological pressure, which is what ‘yali’ means and is also what the Cohen scale measures, and so it is applicable to Chinese culture. Among the participants, 42% mentioned economic-related stress, in particular stress related to paying rent or a mortgage. A few specifically mentioned their responsibility to provide for their families and how hard it was to make money. Others talked about work-related stress, such as business being difficult with fierce competition. Children’s education was also a source of stress for migrants with preschool-aged children. As Shanghai had been shutting down schools for migrants’ children, parents are faced with a highly precarious policy environment regarding whether their children can receive an education in Shanghai. When asked what they do to relieve stress, two-thirds of respondents indicated that they relaxed at home, either watching TV or sleeping. Among them, some also had a second way, including doing sports, talking to friends/family members or engaging in online discussions. Only 10% considered group activity as a key measure for coping with stress, such as having a meal out and/or drinking with friends or going to a movie together. When asked whether there is a small social group that could give them a sense of belonging or well-being, one-third of respondents gave a negative answer. Others indicated that such a social group comprised either family, friends or colleagues, but five people suggested that it was the online community rather than people in their real lives that gave them a sense of belonging. At least six participants mentioned that playing cards was the group activity that gave them a sense of involvement and well-being, which is consistent with the finding in our ethnographic study (L. Richaud and A. Amin, manuscript submitted).

In this pilot survey, we also used conventional instruments to measure migrants’ stress and mental health. Perceived stress was measured by a 10-item perceived stress scale (PSS-10), which was developed by Cohen, Kamarack and Mermelstein^25^ to tap into the degree to which respondents found their lives unpredictable, uncontrollable and overloaded. It asks participants how often they had certain thoughts or feelings, such as feeling upset because of something that happened unexpectedly in the past month. Mental health was evaluated by the six-item Kessler Psychological Distress Scale (K6), which has been validated and widely used in China.^26^ K6 is a rapid screening instrument for cases of mental illness. The respondents were asked how often they felt (1) nervous, (2) hopeless, (3) restless or fidgety, (4) so depressed that nothing could cheer them up, (5) that everything was an effort and (6) worthless in the past 30 d. The total K6 score ranges from 0 to 24, with a lower score indicating better mental health.

The results show that three-quarters of migrants had moderate stress and half of those surveyed had moderate mental distress. Only two cases showed high perceived stress and three cases potentially indicated severe mental illness. Unfortunately, the sample size is too small to perform any meaningful quantitative analysis, but the ethnography-informed survey provided rich data on migrants’ everyday experience and on stress in the city, which could complement the knowledge on the association between city living and mental health. It enabled us to bring in some of the issues from the ethnography, particularly on the feelings and perceptions that migrants had towards the city of Shanghai and their lives in Shanghai, the sources of stress in their daily lives and their strategies for coping with stress. The survey also successfully dug deeper into their social experiences, such as access to medical insurance, previous work and life experiences before coming to Shanghai, separation from family and small social groups that gave them a sense of belonging and well-being. The survey also incorporated other items that we have not discussed in this paper due to limitations of space, including social capital and childhood adverse experience scales. From the pilot survey experience, open questions could capture more feelings and thoughts of respondents (such as ‘Why do you (dis)like Shanghai?’) than multiple choice questions, but some respondents did not have the patience or the ability to formulate their answers. If used for a bigger sample survey, it is recommended to reduce the length of the survey and avoid too many open questions.

## New survey method through smartphone app

While the existing literature on the interactions between the urban environment and mental health provides valuable insights, it is also constrained by a number of methodological limitations.^27,28^ First, the vast majority of studies have used data with coarse geographical resolution (e.g. at borough level) and therefore we were unable to isolate specific features of the built and social environment that might be responsible for the emergence of mental illness. Second, most studies have used a cross-sectional design involving the acquisition of a single ‘snapshot’, without accounting for the fact that people experience a diverse range of urban environments throughout the day. Third, the vast majority of previous studies have been reliant on retrospective assessments that are subject to recall bias.

**Figure 2.**
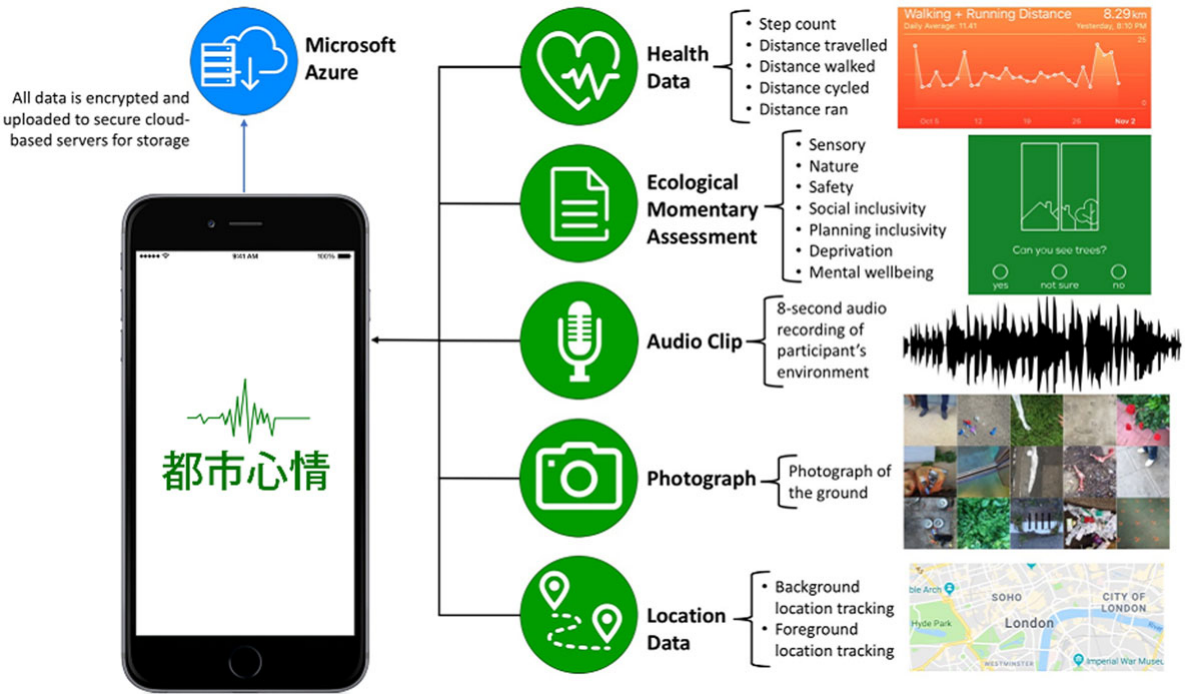
Types of data collected via the Urban Mind app.

In order to help address some of these limitations, we have developed a smartphone app named Urban Mind that monitors the impact of the surrounding environment on mental well-being in real time (see Figure 2). The app was conceived and developed in collaboration with the arts foundation Nomad Projects (http://nomadprojects.org/) and landscape architects J&L Gibbons (https://jlg-london.com/), and was built using the Ionic cross-platform development framework by technical providers Artists & Engineers (http://artistsandengineers.co.uk/). We also developed back-end server software to communicate with the app and a public-facing, project-related website providing information on the research to potential participants. The Urban Mind app can be downloaded for free from the Apple App Store and the Google Play Store, and is available in multiple languages including English, Mandarin, Cantonese, French and German. Participation in the project is anonymous as we do not collect identifiable information (e.g. names, addresses and telephone numbers) from the people who take part in the study. After downloading and installing the app on their smartphones, participants are presented with a brief description of the aim and methodology of the project and are asked to provide informed consent (please refer to the private policy page at https://www.urbanmind.info/privacy-policy#p2, which explains what information is being collected, how it is stored and how long it will be preserved). Once informed consent is granted, data collection can start.

The Urban Mind app (https://www.urbanmind.info) collects data using a methodology known as ecological momentary assessment, which involves repeated sampling of individuals’ current experiences in real-time and real-world contexts.^29^ Several previous studies have used ecological momentary assessments to assess mental health issues.^30–33^ The use of a smartphone-based ecological momentary assessment has three critical benefits: first, it minimizes recall bias as the data are collected in real time; second, it maximizes ecological validity as data are collected in real-world environments; third, it allows multiple measurements over time, providing insight into dynamic changes in mental state and/or behaviour that may not be captured by a single snapshot. Data collection via the Urban Mind app include the following.

**Figure 3.**
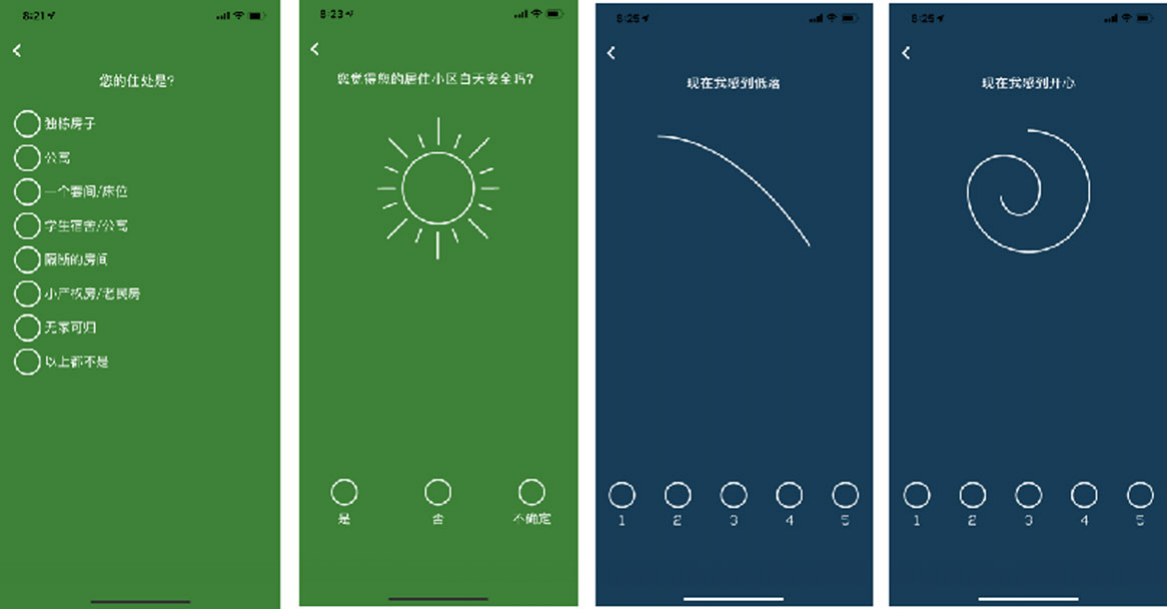
Screenshots of the Chinese versions of the Urban Mind app.

1. A baseline assessment of demographics (e.g. age, gender and ethnicity) and socioeconomics (e.g. education, occupation, housing and working conditions). This information allows us to assess the representativeness of our sample and explore the impact of demographic and socioeconomic variables of interest on our findings.
2. Three ecological momentary assessments per day over a period of 2 wk resulting in a total of 42 ecological momentary assessments. Each ecological momentary assessment takes about 1–2 min to complete and covers the following: (i) six dimensions of the surrounding environment including sensory stimulation, social inclusivity (i.e. whether a place is easy to navigate and there is somewhere comfortable to sit), feeling of safety, contact with nature, inclusivity from a planning perspective and deprivation; (ii) an individual’s mental well-being including both positive and negative affect; (iii) an individual’s geographical location using global positioning system-based geotagging; (iv) an individual’s so-called ‘health data’ including number of steps, distance walked, distance cycled and distance travelled. When people receive a prompt to complete an ecological momentary assessment, they have 60 min to do so before this is marked as incomplete. This allows participants a time frame in which to respond to the prompt without the need to interrupt any activities they are currently engaged in. If the internet is inaccessible at the time of an ecological momentary assessment, the data are stored on the device and uploaded when mobile or wi-fi internet access is next available. Within each ecological momentary assessment, the vast majority of the questions are associated with distinct icons (see Figure 3). This extensive use of iconography has two advantages: first, it makes the experience of using the app more enjoyable, thereby promoting sustained engagement; second, it shortens the time required to complete an ecological momentary assessment, because users learn to recognize each question from the associated icon.
3. Each time a participant completes an ecological momentary assessment, they are also invited to collect and submit photographic and audio recordings of their surrounding environment. The rationale for inviting participants to document their surrounding environment is twofold: first, these photographic and audio recordings are used to disseminate the research on the project-related website and social media platforms; second, the activity of documenting one’s environment is intended to promote sustained engagement with the tool as well as the wider research project. In addition, we envisage that these photographic and audio recordings could be treated as research data amenable to scientific investigation in the future; for example, geographic information system-based time-graphic methods could be applied to the photographic and audio recordings to generate a geo-narrative account of the data.^34^ A selection of the photographs (https://www.urbanmind.info/data-image) and audio recordings (https://www.urbanmind.info/data-audio) submitted from participants can be seen and heard on our website.

The project has received full ethical approval from the Psychiatry, Nursing and Midwifery Research Ethics Subcommittee at King’s College London (LRS-17/18-6905). As data collection in China has only recently started and is still ongoing, we are unable to present any results at this stage. However, we provide a summary of the data collected from participants up to March 2019 in Table 2. It can be seen that the vast majority of participants are of Chinese Han ethnicity, grew up in a town (10 000–100 000 people) or a city (100 000–1 000 000 people) and now live in a large city (>1 000 000 people). It can also be seen that our sample cannot be considered representative of the general population of China, or even the urban population of Shanghai and Beijing, where the majority of our data come from. This is because our participants tend to be educated to university level and have an average age of just 26.38 y. In addition, our sample was not randomly derived from a specific population because participation is based on having a smartphone and choosing to download and use our app. Moving forward, we plan to recruit a more diverse sample and investigate how the results change as a function of demographic and socioeconomic factors. This could be achieved, for example, through the use of ‘ambassadors’ from a wide range of demographic and socioeconomic backgrounds, who could disseminate the project and promote participation among their peers and communities. In addition, we plan to model the information on demographics, socioeconomics and histories of mental health in our statistical models in order to explore the impact of these variables on the findings within the final sample. The point of this work is ‘proof of principle’ that we can recruit respondents in Shanghai, and that we can receive and use the data. Our next steps will be to extend the Shanghai sample, to use the app in Sao Paulo and Toronto and then hopefully in cities in India and Africa.

**Table 2.** Descriptive statistics of the participants in the Urban Mind project based in China (up to March 2019)

Number of participants	116
Gender	
Male	45 (38.8%)
Female	71 (61.2%)
Age, y	mean: 26.38; SD: 7.03; minimum: 16; maximum: 59
Ethnicity	
Chinese Han	109 (94.0%)
Chinese non-Han	4 (3.4%)
African	0 (0.0%)
Caribbean	1 (0.9%)
Caucasian	0 (0.0%)
East Asian (other than Chinese)	0 (0.0%)
South Asian	0 (0.0%)
Latino/Hispanic	0 (0.0%)
Middle Eastern	0 (0.0%)
Mixed	0 (0.0%)
Other	2 (1.7%)
Where did you grow up?	
In a large city (>1 000 000 people)	11 (9.5%)
In a city (100 000–1 000 000 people)	45 (38.8%)
In a town (10 000–100 000 people)	41 (35.3%)
In a village (<10 000 people)	13 (11.2%)
Multiple places	6 (5.2%)
Where do you live now?	
In a large city (>1 000 000 people)	69 (59.5%)
In a city (100 000–1 000 000 people)	28 (24.1%)
In a town (10 000–100 000 people)	17 (14.7%)
In a village (<10 000 people)	2 (1.7%)
Educational level	
University	99 (85.3%)
Apprenticeship/technical training	9 (7.8%)
High school	8 (6.9%)
Less than high school	0 (0.0%)
Occupation	
Student	78 (67.2%)
Employed	33 (28.4%)
Self-employed	4 (3.4%)
Retired	1 (0.9%)
Unemployed	0 (0.0%)
Where do you live?	
Houses built on rural land/farmers’ old houses	4 (3.4%)
Flat	42 (36.2%)
House	5 (4.3%)
Student residence	52 (44.8%)
Studio/bedsit	11 (9.5%)
Who do you live with?	
Alone	19 (16.4%)
Family	38 (32.8%)
Friends	33 (28.5%)
Acquaintances	4 (3.5%)
Colleague	13 (11.2%)
Strangers	3 (2.6%)
None of the above	19 (16.4%)
Do you feel safe where you live during the day?	
Yes	110 (94.8%)
No	2 (1.7%)
Not sure	4 (3.5%)
Do you feel safe where you live at night?	
Yes	94 (81.0%)
No	7 (6.0%)
Not sure	15 (12.9%)
Is it clean and well looked after where you live?	
Yes	93 (80.2%)
No	14 (12.1%)
Not sure	9 (7.8%)
Are there derelict buildings near where you live?	
Yes	28 (24.1%)
No	80 (69.0%)
Not sure	8 (6.9%)
Do you feel welcome amongst your neighbours?	
Yes	75 (64.7%)
No	4 (3.5%)
Not sure	37 (31.9%)
Do you feel they would be willing to help you?	
Yes	91 (78.5%)
No	5 (4.3%)
Not sure	20 (17.2%)
Do you feel they share the same values as you?	
Yes	34 (29.3%)
No	23 (19.8%)
Not sure	59 (50.9%)
How would you rate your physical health overall?	
Very good	13 (11.2%)
Good	64 (55.2%)
Fair	38 (32.8%)
Poor	1 (0.9%)
Very poor	0 (0.0%)
Not sure	0 (0.0%)
How would you rate your mental health overall?	
Very good	19 (16.4%)
Good	58 (50.0%)
Fair	37 (31.9%)
Poor	1 (0.9%)
Very poor	0 (0.0%)
Not sure	1 (0.9%)
Has a doctor ever diagnosed you with a mental health condition?	
Yes	4 (3.4%)
No	112 (96.6%)
Has a doctor ever diagnosed a close relative of yours with a mental health condition?	
Yes	6 (5.2%)
No	110 (94.8%)
How many hours do you work or study on average per day?	mean: 7.34; SD: 2.95; minimum: 0; maximum: 18
How many people do you live with?	mean: 2.67; SD: 1.05; minimum: 1; maximum: 6
Number of ecological momentary assessments completed	mean: 21.70; SD: 11.00; minimum: 1; maximum: 39

## Conclusions

This paper reports on a significant element of our overall Economic and Social Research Council/Newton-funded project on Mental Health, Migration and the Megacity. The project aimed at deepening the conventional collection of epidemiological data, which we argue fails to capture significant aspects of the mechanisms that connect urban life to mental disorder. We suggest that we need to improve the ways in which sociological analysis and biological analysis might work together through the identification of mechanisms, imagined and confirmed through data, of the way in which urban life gets ‘under the skin’ (see Manning^35^ for a detailed review of these issues). The argument re-examines the use of mechanisms in scientific explanation as a basis for identifying the shortcomings of current epidemiology, and to suggest the possibility of a new ‘mechanism-rich’ epidemiology. In this paper we have reported on two examples of our steps towards such a ‘mechanism-rich’ epidemiology, based on fieldwork in Shanghai, which we explicitly set up as a means of developing new approaches. Such experience-based approaches are capable of grasping bodily, sensory, emotional and spatial dimensions of urban life and therefore have distinct advantages in studying urban psychosis nexus. This has been clearly demonstrated in other studies. For example, using visual methodologies such as videos and go-alongs, Söderström and colleagues captured the aspects of the urban milieu that are perceived as stressful to patients with psychotic disorders as well as their dealing tactics.^36,37^ Using ethnography, Bister, Klausner and Niewöhner^38^ examined the ‘niching’ of people living with a psychiatric diagnosis—how they render urban assemblages habitable—to develop a mode of dwelling that is bearable. In particular, Winz^39^ proposed an atmospheric approach, stressing sensory perception, to study minor stress events and their accumulation in cities. Compared with quantitative methods or standard interviews, these approaches provide distinctive information in that they are able to provide insight into urban living as an embodied and mobile experience of space and place, beyond verbal representation.^36^ The two methods proposed in this paper could in turn be beneficial to traditional ethnography, in that working alongside epidemiological surveys could push anthropologists towards focused exploration and explanation by adding a qualitatively different kind of analytical slant to the ethnographic approach.^22^

It is also important to acknowledge that our methodological approach has a number of limitations. With respect to the use of our ‘deep’ surveying instrument, the pursuit of a mechanism-rich epidemiology means that we are inevitably dependent on an increase in the time-consuming use of detailed interviews, which either requires greater resources or restrictions with regard to sample size. With respect to the use of a smartphone app to monitor participants in real time, there are at least five limitations to consider. First, because the data are acquired using an observational rather than an experimental design, it is not possible to establish whether the observed associations reflect a direct causal impact; second, the use of an ecological momentary assessment relies on self-reports, which are known to be prone to potential bias^29^; third, our sample comprised smartphone users with a higher than average level of education and an average age of just 26.38 y, and therefore cannot be considered representative of the general population; finally, it is important to acknowledge that mental well-being and mental health are not the same thing: mental well-being refers to positive states of being, feeling, thinking and behaving, while mental health incorporates a range of negative and positive states from severe mental illness to excellent mental health. Despite these limitations, we suggest that both of these pilots have demonstrated good feasibility, and we are in the process of developing their application into two new funded research sites, in Sao Paulo and Toronto. An initial paper on the Sao Paolo work is included in this special issue. We anticipate further iterations and development of these instruments in future work planned for Delhi and Lagos, depending on further grant support.
